# Adolescent Tillaux Fractures: A Systematic Review of the Literature

**DOI:** 10.7759/cureus.12860

**Published:** 2021-01-22

**Authors:** Sameem Tak, Mobeen K Qureshi, James A Ackland, Rizwan Arshad, Javed Salim

**Affiliations:** 1 Trauma and Orthopaedics, University Hospitals of Leicester, Leicester, GBR; 2 Trauma and Orthopaedics, East Lancashire NHS Hospitals, Blackburn, GBR; 3 Psychology, University of Cambridge, Cambridge, GBR; 4 Trauma and Orthopaedics, Hull University Teaching Hospitals NHS Trust, Hull, GBR

**Keywords:** tillaux, fracture, ankle, orthopaedic, transitional, adolescent, juvenile

## Abstract

The Tillaux fracture is an uncommon injury to the anterolateral distal tibial epiphysis. It occurs during a distinct time period when adolescent patients are transitioning to skeletal maturity. Owing to its rarity, the optimal management strategy for this fracture is not well-described. The aim of this review was to assess the outcomes of operatively and nonoperatively managed displaced adolescent Tillaux fractures. We analysed articles from The Cochrane Library, PubMed, MEDLINE, and EMBASE databases that met our predetermined inclusion and exclusion criteria according to Preferred Reporting Items for Systematic Reviews and Meta-Analysis (PRISMA) statements. A descriptive data analysis was performed. A total of 461 articles were identified from the data search, of which 13 articles were included for full-text analysis. Five of these studies reported recognised patient outcome measures and the remaining eight reported on radiographic follow-up. The reported studies included a total of 114 patients with Tillaux fractures; 58.8% of patients were female and 34.2% were male. Mean ages ranged from 12.5 to 15 years, with the youngest patient being 12 years old and the oldest 17 years old. Overall mean follow-up was 42.8 months. Of the patients, 40.4% were treated with open reduction internal fixation (ORIF), 14.9% with closed reduction internal fixation (CRIF), and 1.8% arthroscopically. The remainder were treated nonoperatively. Outcome measures were excellent for all patients irrespective of operative management choice. Follow-up radiographic deformity was only evident in Tillaux fractures that were managed nonoperatively; deformity included poor joint congruity, angular deformity, and tibial shortening. These nonoperative patients have a residual fracture displacement of 2 mm. There were no reported instances of premature physeal closure for any patient. This review shows that excellent patient outcomes have been reported for different methods of operative fixation, however, study sizes are small and data is sparse. Further robust comparative studies are required to identify definitive conclusions. The use of established clinical and radiographic outcome measures will help improve the quality of future studies for this relatively rare injury.

## Introduction and background

Sir Astley Cooper first described a fracture of the lateral tibial plafond in 1822 [1]. Paul Jule Tillaux then defined an experimental mechanism for the occurrence of this fracture in 1876, in which the pull of the anterior inferior tibiofibular ligament caused an avulsion fracture of the distal tibia in adult cadavers [2]. Henry Chaput subsequently demonstrated the radiographic appearances of this fracture in 1899 [3]. The Chaput tubercle can be seen as the insertion site of the anterior inferior tibiofibular ligament at the anterolateral aspect of the distal tibia. When fractured, the lesion is most commonly referred to as a Tillaux fracture and occasionally as a Tillaux-Chaput fracture. The initial description of this injury was in adults, with occurrence in the adolescent population being referred to as the juvenile Tillaux fracture. In 1964, Kleiger and Mankin were the first to report on a series of adolescent patients with this injury [4].

Both Tillaux and Triplane fractures of the distal tibial epiphysis are referred to as transitional fractures because they occur during an 18-month period of transition from skeletal immaturity to maturity [5]. The distal tibial physis closes between 12 and 17 years in females and 15 and 20 years in males. This closure occurs in a predictable asymmetrical pattern, beginning centrally, then anteromedially, posteromedially and, finally, laterally; whilst the lateral aspect of the physis is open, it is vulnerable to injury [6-10]. The mechanism leading to a Tillaux fracture is usually forced external rotation of the foot, resulting in a Salter-Harris type III epiphyseal injury [11-12].

Tillaux fractures are consequently rarely seen in adults since the anterior inferior tibiofibular ligament is more likely to rupture than to avulse a bony fragment at its attachment site [13-14]. The incidence of Tillaux fractures is 2.9% of juvenile epiphyseal growth plate injuries [15]. Radiographic workup includes anteroposterior, lateral and mortise view radiographs [16]. Computed tomography )CT) scans have been shown to be more sensitive in detecting Tillaux fracture displacement >2 mm compared to radiographs [17].

Since these injuries are intra-articular with growth plate involvement, the surgeon’s aim is to achieve an anatomic reduction of the joint surface to minimise the risk of post-traumatic arthritis, pain and stiffness [18]. Treatment strategy depends on displacement; minimally displaced fractures (<2 mm) are often treated non-operatively by cast immobilisation, and displacement ≥2 mm is generally an indication for operative repair [9-10].

There is limited data to support a consensus on displaced Tillaux fracture management. Reports in the literature often group Tillaux and triplane fracture data together (the transitional fractures) and many studies are limited by small sample size or lack of validated functional outcome measure use. The aim of this paper was to review the outcomes of operatively and nonoperatively managed displaced adolescent Tillaux fractures.

## Review

Methods: search strategy 

An online systematic literature search in accordance with the Preferred Reporting Items for Systematic Reviews and Meta-Analyses (PRISMA) guidelines was performed. The Cochrane Library was searched, and the National Institute for Health and Care Excellence (NICE) healthcare database advanced search (HDAS) was utilised via OpenAthens to search the PubMed, MEDLINE and EMBASE databases. The search was performed using Boolean operators and the wildcard symbol (*) to truncate search terms for Tillaux OR Tillaux-Chaput OR distal tibia* epiphys* OR anterolateral distal tibial OR transitional OR Kleiger AND fracture* AND child* OR teen* OR adolescen* OR juvenile. The databases were searched from their inception until October 2020. All reference lists of included articles were trawled for further relevant studies missed by the search. EndNote version X9 (Thomas Reuters, New York City, NY) was used to organise the database searches and to filter duplicate articles. Unpublished work was not sought.

Methods: eligibility criteria

Studies were included if they reported: any original article, age <18 years old with an isolated Tillaux fracture of any management strategy, such as nonoperative, open reduction internal fixation (ORIF), closed reduction internal fixation (CRIF) or arthroscopic, and stated follow-up with a standardised patient outcome measure such as American Orthopaedic Foot & Ankle Society (AOFAS), Foot and Ankle Outcome Score (FAOS), Modified Weber Protocol (MWP) [19-22] or radiographic follow-up (union rate, premature physeal closure, fracture healing, joint incongruity, angular deformity).

Studies that were excluded were: case reports, case series <2 patients, review articles, basic science articles, patients aged >18 years old, triplane fractures, cadaveric studies, Tillaux fractures with an associated lower limb injury, studies reporting Salter-Harris III fractures without explicit identification of Tillaux fractures, reports with unstandardised descriptive follow-up and incomplete data sets. The language of publication was limited to English.

Methods: data extraction and statistical analysis 

Following initial duplication removal, titles and abstracts for 340 articles identified through the database search were screened independently by two authors against the inclusion and exclusion criteria. This was achieved in a parallel blinded manner by using the Rayyan QCRI tool for screening abstracts [23]. Where there was uncertainty on an article's suitability for inclusion, full texts were obtained and reviewed. There was no disagreement in study selection between authors.

The following data were extracted: demographics for the number of patients, age, gender, indication for surgery, fracture management choice, outcome measure, radiographic outcome and follow-up length of time. Appendix A shows the quality assessment tool that was used to assess the risk of bias of the included studies, adapted from Murad et al. for case series and ROBINS-I for non-randomised interventional studies [24-25]. All continuous data were pooled and descriptive data analysis performed.

Results

**Figure 1:**
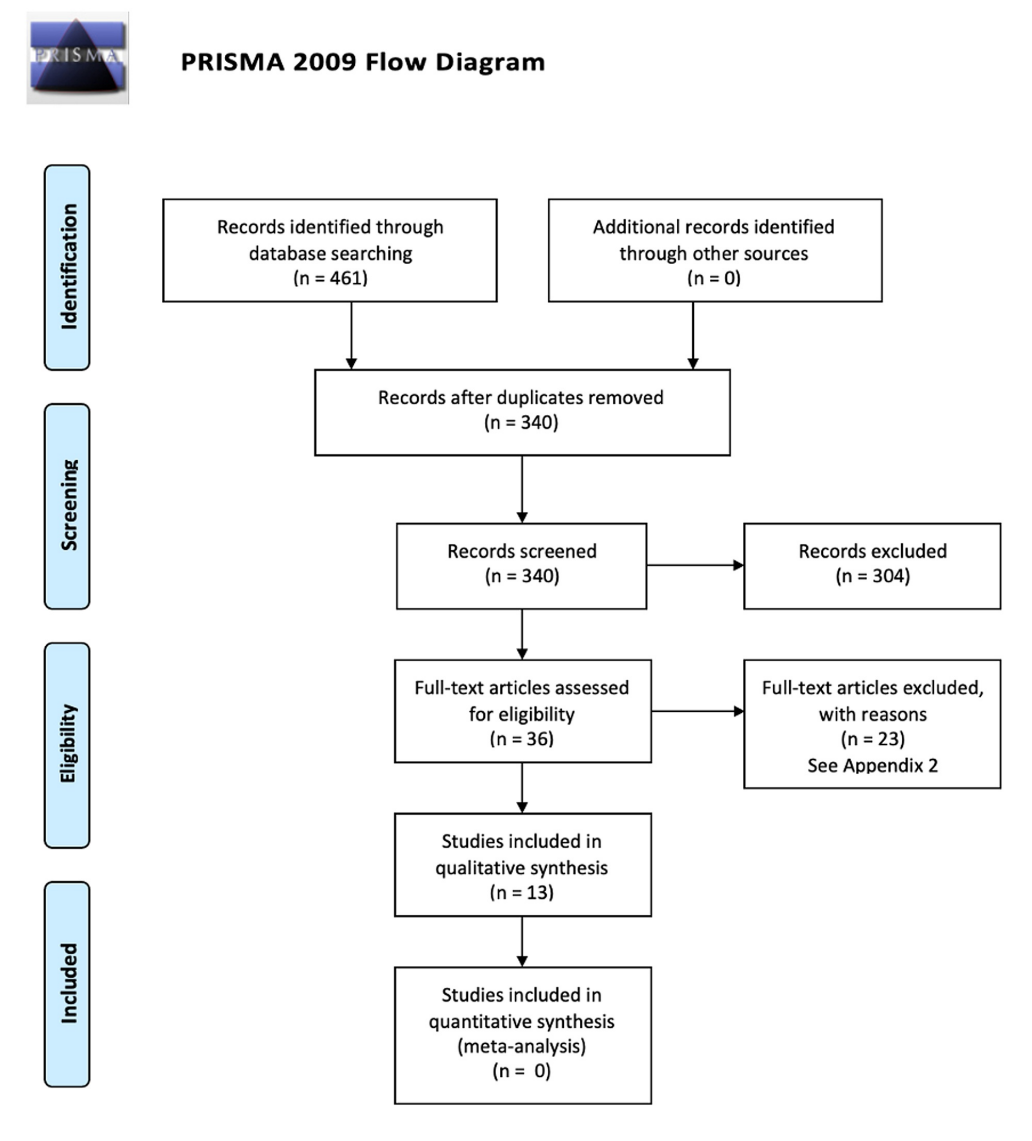
Flow diagram of Preferred Reporting Items for Systematic Reviews and Meta-Analyses (PRISMA) strategy used to determine eligible studies

The preliminary literature search yielded 461 articles, of which 13 were put forward for analysis. Figure 1 shows the PRISMA flowchart for study selection. No further unique articles were found from trawling citations of included articles. Twenty-three full-text articles were deemed ineligible for inclusion in the review; the reasons for their exclusion are summarised in Appendix B.

Only five of the 13 included articles used substantiated patient-reported outcome measures [26-30]. The remaining eight articles reported radiographic follow-up only [11-12,31-36]. The included studies were either case series or uncontrolled cohort studies.

Table 1 summarises the characteristics of the studies included in our analysis. The 13 studies contributed a total of 114 patients, 58.8% (n=67) of which were female and 34.2% (n=39) of which were male. Mean ages ranged from 12.5 to 15 years, with the youngest patient being 12 years old and the oldest 17 years old. Twelve of the 13 studies reported a mean follow-up period; the overall mean follow-up was 42.8 months, ranging from six months to 27 years.

**Table 1:** Study characteristics Note: Unrecorded data are represented by '-'

Author	Year	Study design	Number of patients	Gender male/female	Mean age (years)	Age range (years)	Indication for surgery	Mean follow up (months)	Follow up range (months)
Al-Ashhab & Mohamed [26]	2019	single-arm, uncontrolled cohort study	13	4/9	13.4	12-17	>2mm fracture displacement	41.7	24-60
Kim et al. [27]	2010	case series	2	2/0	12.5	12-13	-	15	12-18
Tiefenbock et al. [28]	2016	case series	7	4/3	15	14-16	>2mm fracture displacement	79	40.43-126.80
Feng et al. [29]	2018	case series	2	1/1	13	10-16	>2mm fracture displacement	17.5	15-20
Kaya et al. [30]	2007	case series	10	4/6	13.1	12-14	2mm or more fracture displacement	54	32-75
Gourineni & Gupta [33]	2011	case series	8	-	12.9	10-14	>1mm fracture displacement	12	-
Leary et al. [35]	2009	case series	26	12/14	13.5	-	>2mm fracture displacement	8.47	-
Dailiana et al. [31]	1999	case series	3	2/1	14	13-15	-	56	18-102
Stefanich & Lozman [11]	1986	case series	5	0/5	12.8	12-15	2mm or more fracture displacement	64.8	12-108
Landin et al. [34]	1986	case series	17	3/14	13.9	12-16	-	112.2	36-324
Dias & Giegerich [32]	1983	case series	9	3/6	13.5	12-14	>2mm fracture displacement	-	18-36
Spiegel et al. [12]	1978	case series	6	3/3	13.5	-	all nonoperative	40.8	-
Molster et al. [36]	1977	case series	6	1/5	14.7	13-16	moderate vs. minimal displacement, not quantified	12.2	6-21

In total, 40.4% (n=46) of patients were treated with ORIF, 14.9% (n=17) with CRIF and 1.8% (n=2) arthroscopically. Four point four percent (4.4%; n=5) did not specify the operative treatment and 38.6% (n=44) of patients were treated nonoperatively.

Table 2 shows the five studies that used recognised standardised patient outcome measure scores; three used AOFAS, one used the MWP and one used FAOS. Within these five studies, 55.9% (n=19) were ORIF, 38.2% (n=13) were CRIF and 5.9% (n=2) were arthroscopic. All five of these studies reported excellent scores for all patients regardless of operative management choice.

**Table 2:** Five studies that used standardised patient outcome measures ORIF, open reduction internal fixation; CRIF, closed reduction internal fixation; AOFAS, American Orthopaedic Foot & Ankle Society; MWP, Modified Weber Protocol

Author	Year	ORIF	CRIF	Arthroscopic	Outcome measure	Cast immobilisation length
Al-Ashhab & Mohamed [26]	2019	3	10	-	mean AOFAS 97 (range 95-100)	6 weeks NWB
Kim et al. [27]	2010	-	2	-	mean MWP Excellent	-
Tiefenbock et al. [28]	2016	6	1	-	mean foot and ankle score 98.71	4-8 weeks
Feng et al. [29]	2018	-	-	2	mean AOFAS 92	6 weeks NWB
Kaya et al. [30]	2007	10	-	-	mean AOFAS 99.3 (range 97-100)	6 weeks

Table 3 shows the 80 patients with radiographic follow-up. All patients treated with either ORIF (33.8%, n=27) or CRIF (5%, n=4) showed excellent radiographic follow-up outcomes. Four patients who were treated nonoperatively had radiographic deformity at follow-up, including joint incongruity, tibial shortening, valgus deformity and angulation. Three of these patients had no attempt at fracture reduction performed and had a residual gap of 2 mm fracture displacement [34]. The authors of the patient with joint incongruity did not comment upon fracture displacement [12].

**Table 3:** Eight studies that reported on the radiographic follow-up of Tillaux fractures Unrecorded data are represented by '-' ORIF, open reduction internal fixation; CRIF, closed reduction internal fixation; PPC, premature physeal closure

Author	Year	Nonoperative	ORIF	CRIF	Arthroscopic	Operative unspecified	Radiographic follow-up	Cast immobilisation length
Gourineni & Gupta [33]	2011	-	5	3	-	-	all fractures healed radiographically within 4 weeks	4 weeks
Leary et al. [35]	2009	21	-	-	-	5	0% of radiographic PPC	-
Dailiana et al. [31]	1999	-	3	-	-	-	all fractures healed radiographically	4 weeks
Stefanich & Lozman [11]	1986	1	4	-	-	-	all fractures healed anatomically	6-9 weeks
Landin et al. [34]	1986	10	6	1	-	-	ORIF: normal CRIF: normal no reduction: 3 cases with radiographic abnormality (1: valgus deformity of 5 degrees and 17mm tibial shortening, 2: 5 degrees of anterior angulation, 3: 6 degrees of dorsal angulation) all 3 had residual fracture displacement of 2mm	4-6 weeks
Dias & Giegerich [32]	1983	5	4	-	-	-	all fractures healed radiographically	6 weeks
Spiegel et al. [12]	1978	6	-	-	-	-	1 x joint incongruity (only 4/6 pts for follow up)	-
Molster et al. [36]	1977	1	5	-	-	-	no radiographic deformity	5-7 weeks

Discussion

The adolescent Tillaux fracture is an uncommon injury and, subsequently, there is a lack of high-quality evidence in the literature. The available data is largely confined to sporadic case reports and small number case series. Data are often presented as a combination of Tillaux and triplane fractures, with many reports lacking robust follow up (see Appendix B) [18,27,33].

Excellent outcomes were seen irrespective of operative modality (ORIF vs. CRIF vs. arthroscopic) in studies reporting patient outcome measures. All five of these studies had patients with 2 mm or more of fracture displacement as an indication for surgery. Anatomic reduction without persistent articular incongruity in Tillaux fractures appears to be associated with favourable outcomes. Whilst an initial attempt at closed reduction is warranted, it may not always be possible to obtain the reduction; periosteal interposition at the fracture site may prevent reduction and necessitate definitive open reduction. Given the small numbers of patients, it is important to consider that the available literature may not be representative of the real-world Tillaux population; reporting bias may take effect if operative patients with unfavourable outcomes are not reported upon. In addition to this, the mean length of follow-up for patients with reported outcome measures was 3.4 years, which may overlook long-term complications.

It has been suggested that whilst boys have a higher occurrence rate of epiphyseal fractures, the subgroup of Tillaux fractures occurs more frequently in girls [11,37-38]. Our results support this observation, with 58.8% (n=67) of Tillaux fractures occurring in girls.

A quantitative meta-analysis was considered to provide statistical evidence for the comparative efficacy of each type of operative treatment (ORIF, CRIF, arthroscopic). Since there were no recorded cases of adverse radiographic findings for any operative treatment type, only patient outcome measures could feasibly be used as a dependent variable in quantitative analysis. Unfortunately, the total sample for studies recording a patient outcome measure (n=34) was insufficient for the statistical discovery of even relatively large effects. This is further confounded by the presence of a ceiling effect within the distribution of outcome measure scores, indicative of a high success rate amongst all treatment types. The scarcity of cases is a function of the rarity of Tillaux fractures, but from the small quantity of data available, a descriptive review shows all three operative treatment types to be associated with excellent outcomes.

If fracture displacement persists at 2 mm or more, it should be reduced. Eighty patients had radiographic follow-up reported, and four patients had poor radiographic sequelae. All four of these patients were treated nonoperatively, with three of them having had no attempt at reduction. These three patients had a residual fracture displacement of 2 mm. The abnormalities included poor joint congruity, angular deformity and shortening. There were no reported instances of premature physeal closure in our data. By nature of the demographic of Tillaux fractures, growth arrest appears to be less likely to occur since this injury occurs in older adolescent patients who are close to skeletal maturity [39].

## Conclusions

The adolescent Tillaux fracture is an uncommon fracture to the lateral distal tibial epiphysis. Since these fractures are intraarticular, adequate anatomical reduction should be the primary goal, irrespective of the operative approach. Excellent patient outcomes have been reported for different methods of operative fixation, however, study sizes are small, and data is sparse. Further comparative studies are required to identify definitive conclusions. The use of established clinical and radiographic outcome measures will help improve the quality of future studies for this relatively rare injury.

## Appendices

Appendix A

**Table 4:** Quality assessment tool used to assess the risk of bias of the included studies

Study	Risk of bias	Comment
Al-Ashhab & Mohamed [26]	Moderate	Presented as a cohort but only single arm with no comparative group
Kim et al. [27]	Low	-
Tiefenbock et al. [28]	Low	Limited follow-up data (only five of seven available for long term follow-up)
Feng et al. [29]	Low	-
Kaya et al. [30]	High	Unclear selection approach, undefined time period
Gourineni & Gupta [33]	Moderate	Comparatively short follow-up period
Leary et al. [35]	Moderate	Operative intervention insufficiently reported
Dailiana et al. [31]	Moderate	Unclear intervention criteria; did not specify fracture displacement requirement for surgery
Stefanich & Lozman [11]	High	Unclear selection approach, undefined time period
Landin et al. [34]	High	Selection bias; only 59 of 79 patients came forward for examination
Dias & Giegerich [32]	Moderate	Radiographic outcome analysis insufficiently defined (criteria for radiographic evaluation not defined)
Spiegel et al. [12]	Moderate	Inadequate follow-up data (only four of six patients had follow-up outcomes recorded)
Molster et al. [36]	High	Unclear selection approach, comparatively short follow-up period, radiographic outcome analysis insufficiently defined, unclear intervention criteria; did not specify fracture displacement requirement for surgery (defined displacement as moderate or minimal)

Appendix B

**Table 5:** List of full-text articles excluded with reasons

Article	Year	Journal	Reason for exclusion
Lurie et al. 2020	2020	The Journal of Bone and Joint Surgery	Grouped Tillaux and triplane together for functional outcome assessment
Stenroos et al. 2019	2019	Acta Orthopaedica	Only one patient available for follow-up in Tillaux group
Zelenty et al. 2018	2018	Journal of Pediatric Orthopaedics	Could not identify Tillaux group – mixed with triplane
Aguilar Ezquerra et al. 2017	2017	Revista de la Facultad de Ciencias Medicas	Foreign language
Choudhry et al. 2014	2014	Journal of Pediatric Orthopaedics	Grouped Tillaux and triplane fractures
Liporace et al. 2012	2012	Orthopedics	Compared imaging choices, no relevant follow-up
Strohm et al. 2011	2011	Acta chirurgiae orthopaedicae et traumatologiae Cechoslovaca	Grouped Tillaux and triplane fractures
Castellani et al. 2009	2009	The Journal of Trauma	Could not identify a Tillaux cohort from the data
Panagopoulos et al. 2008	2008	Injury	Letter to editor
Jennings et al. 2007	2007	The Journal of Foot and Ankle Surgery	Only one Tillaux patient in a case series of six patients
Pannier et al. 2006	2006	Revue de chirurgie orthopedique et reparatrice de l'appareil moteur	Foreign language
Nenopoulos et al. 2005	2005	Journal of Pediatric Orthopaedics	Could not assign follow-up to fracture group
Weinberg et al. 2005	2005	Injury	Grouped treatment outcomes for all transitional fractures
Horn et al. 2001	2001	Journal of Pediatric Orthopaedics	Cadaveric study
Schlesinger et al. 1993	1993	Journal of Pediatric Orthopaedics	Insufficient detail on follow-up and follow-up assessment
Melchior et al. 1990	1990	Chirurgie Pediatrique	Foreign language
Felman, AH 1989	1989	Pediatric Radiology	Review article
Von Laer, L 1985	1985	The Journal of Bone and Joint Surgery	Could not assign radiographic follow-up to fracture group
MacNealy et al. 1982	1982	American Journal of Roentgenology	Insufficient detail on follow-up and follow-up assessment
Letts, RM 1982	1982	Journal of Pediatric Orthopaedics	Insufficient detail on follow-up and follow-up assessment
Kump, WL 1966	1966	American Journal of Roentgenology	Insufficient detail on follow-up and follow-up assessment
Crenshaw, AH 1965	1965	Clinical Orthopaedics and Related Research	Could not identify Tillaux patients
Kleiger, B & Mankin, HJ 1964	1964	The Journal of Bone and Joint Surgery	Insufficient detail on follow-up and follow-up assessment
